# Female RNA concussion (FeRNAC) study: assessing hormone profiles and salivary RNA in females with concussion by emergency departments in New Zealand: a study protocol

**DOI:** 10.1186/s12883-024-03653-9

**Published:** 2024-05-02

**Authors:** Natalie Hardaker, Doug King, Patria A. Hume, Tom Stewart, Stacy Sims, Indira Basu, Blair Shilton, James Selfe

**Affiliations:** 1 Faculty of Health and Environmental Science, Sports Performance Research Institute New Zealand (SPRINZ), Auckland University of Technology, New Zealand Wellington, New Zealand; 2 Accident Compensation Corporation, Wellington, New Zealand; 3 Traumatic Brain Injury Network (TBIN), Auckland University of Technology, Auckland, New Zealand; 4 Auckland Bioengineering Institute, The University of Auckland, Auckland, New Zealand; 5 Stanford Lifestyle Medicine, Stanford University, Palo Alto, CA USA; 6 Department of Sport and Exercise Sciences, Wolfson Research Institute for Health and Wellbeing, Durham University, Durham, UK; 7 Technology and Policy Lab - Law School, The University of Western Australia, Perth, Australia; 8 Labtests, Auckland, New Zealand; 9 Department of Health Professions, Faculty of Health and Education, Manchester Metropolitan University, Manchester, UK

**Keywords:** Females, Concussion, Sex hormones, Saliva, Biomarkers, sncRNAs, Recovery, Outcomes

## Abstract

**Background:**

Females of reproductive age with concussion report a greater number of symptoms that can be more severe and continue for longer than age matched males. Underlying mechanisms for sex differences are not well understood. Short non-coding Ribonucleic Acids (sncRNAs) are candidate salivary biomarkers for concussion and have been studied primarily in male athletes. Female sex hormones influence expression of these biomarkers, and it remains unclear whether a similar pattern of sncRNA expression would be observed in females following concussion. This study aims to evaluate recovery time, the ratio of salivary sncRNAs and symptom severity across different hormone profiles in females presenting to emergency departments (ED) with concussion and, to investigate the presence of low energy availability (LEA) as a potential modifier of concussion symptoms.

**Methods:**

This prospective cohort study recruits participants from New Zealand EDs who are biologically female, of reproductive age (16–50 years) and with a confirmed diagnosis of concussion from an ED healthcare professional. Participants are excluded by ED healthcare professionals from study recruitment as part of initial routine assessment if they have a pre-diagnosed psychiatric condition, neurological condition (i.e., epilepsy, cerebral palsy) or more than three previously diagnosed concussions. Participants provide a saliva sample for measurement of sncRNA’s, and online survey responses relating to hormone profile and symptom recovery at 7-day intervals after injury until they report a full return to work/study. The study is being performed in accordance with ethical standards of the Declaration of Helsinki with ethics approval obtained from the Health and Disability Ethics Committee (HDEC #2021 EXP 11655), Auckland University of Technology Ethics Committee (AUTEC #22/110) and locality consent through Wellington hospital research office.

**Discussion:**

If saliva samples confirm presence of sncRNAs in females with concussion, it will provide evidence of the potential of saliva sampling as an objective tool to aid in diagnosis of, and confirmation of recovery from, concussion. Findings will determine whether expression of sncRNAs is influenced by steroid hormones in females and may outline the need for sex specific application and interpretation of sncRNAs as a clinical and/or research tool.

**Trial registration:**

Australian New Zealand Clinical Trials Registry (ANZCTR) registration number ACTRN12623001129673.

## Background

Concussion, as a subset of mild traumatic brain injury, is a brain injury induced by direct or indirect biomechanical force being transmitted to the head via a blow to the head or the body. Disturbance in cellular homeostasis in the brain initiates complex biochemical and neurometabolic changes [1] resulting in a transient ‘energy crisis’ in the brain. Concussion is typically considered a functional rather than structural injury and presents as a variable cluster of physical, cognitive, emotional, and sleep related symptoms. Sports Related Concussion (SRC) has become almost synonymous with male collision sport and has in recent years received increased profile and greater awareness generated through traditional and social media. However, concussion occurs in many activities and environments with data showing that sports collectively account for only 20–40% of all concussions. [2, 3] Motor vehicle accidents and military combat are other common mechanisms of injury in males, [4] whereas females have tended to sustain concussion from falls or incidents of intimate partner violence. [4] As greater numbers of females enter the armed forces and play collision sports, the incidence of concussion in these environments will likely increase. There is a need to ensure that both sport and non-sports related concussion are addressed in research and clinical settings, and in public awareness and media campaigns.

Sex differences are evident in concussion. [5–14] Females often have more severe symptoms that are longer in duration. [15–18] Studies in female athletes also show that symptom endorsement at baseline (i.e., when there is no injury) is more prevalent than that seen in males. [19] Underlying mechanisms for these observed sex differences are not yet well understood and sex as a biological variable in injury recovery remains understudied. [20] Limited data show that sex differences in concussion outcomes (i.e., severity and duration of symptoms) first appear during adolescence, are evident throughout the reproductive years, but that postmenopausal women have similar or better outcomes than men. [5] Women have a considerable physiologic change when transitioning between these life stages with altered levels of estrogen and progesterone. Bi-directional relationships between female sex hormones and concussion could influence symptoms and recovery outcomes. [4, 5, 21, 22] Primary female sex hormones, estrogen and progesterone are typically associated with reproduction, but these hormones are powerful biochemical messengers that influence every body system including, temperature regulation, macronutrient metabolism, hydration, central nervous system fatigue, and brain bioenergetics. [23–26] During reproductive years, estrogen and progesterone fluctuate across the menstrual cycle, which typically lasts 21–35 days. [27, 28] Hormone related symptoms have significant overlap with those symptoms associated with concussion and are similarly considered across physical, cognitive, emotional and sleep related domains. Low Energy Availability (LEA) occurs when dietary energy intake is insufficient to support energy expended in exercise and results in inadequate energy to support the functions required by the body to maintain optimal health; prolonged LEA is a clinical concern that disproportionately affects females, can disrupt endocrine function [29, 30] and presents with a similar set of symptoms to concussion. The prevalence of LEA ranges from 33.5% in recreationally active (tier 2 [31]) females [32] to 59% in sub-elite [33] (tier 3 [31]) and 88% in professional female football players [34] and could be a potential modifier in concussion. Female specific research is therefore needed to understand how sex hormones interact with concussion and to what extent concussion may impact hormone regulation and symptoms.

Diagnosis of concussion may not always be clear; assessment typically includes the use of neurocognitive and physical tests and subjective symptom report. It is also important to identify a plausible mechanism of injury, which can also be difficult to recognise in cases where there are multiple injuries. There are currently no validated tools or tests that can confirm a diagnosis of concussion, this relies on clinical judgement. Recent research in clinical settings and on sports field sidelines [35–45] indicated potential blood biomarkers that may be specific to concussion, including beta-amyloid protein, ubiquitin carboxy-terminal hydro-lase L1 (UCH-L1), glial fibrillar acidic protein (GFAP), S100β, Tau, neurofilament light protein (NFL) and brain derived neurotrophic factor (BDNF). [36–43]. However, due to relatively small heterogenous sample sizes and varying methodological approaches there have been inconsistent research findings and a lack of clarity on the time course or presence of these biomarkers in relation to injury. More recent advances in technologies have enabled investigation into ribonucleic acid (RNA) species as potential biomarkers of disease. [35, 37, 38, 41, 46] MicroRNAs (miRNAs, miRs) act at the post-transcriptional level to regulate protein synthesis and belong to the family of small non-coding RNAs (sncRNAs) (20 to 200 nucleotides in length). MiRNA signatures specific to traumatic brain injury (TBI) including concussion have been reported in blood, cerebrospinal fluid and saliva, and these signatures appear to vary according to TBI severity. Different ‘families’ of miRNA’s may be upregulated or downregulated in response to various conditions (e.g., post-exercise or injury) or disease states.

Non-invasive saliva sampling enables rapid collection of data immediately after injury and at specified time points thereafter, with the participant able to collect the sample independently. In a recent study [35] utilising a saliva collection protocol, a panel of 14 different sncRNAs present in saliva accurately discriminated between clinical diagnosis or absence of concussion in 156 professional male rugby union players. These sncRNAs have potential to provide objective insight into pathophysiological responses following a concussive injury and may be a useful adjunct as a sideline test and in the clinical environment to aid diagnosis. Findings of studies in male athletes cannot be extrapolated to female populations without further investigation, as expression of sncRNAs in other diseases of the central nervous system is sex dependent. [47–50]

## Methods/design

### Aim

The aims of this study are to: 1) determine if the time to recovery (in days) differs among groups categorised by their hormone profiles at the point of concussion injury, 2) examine whether the ratio of sncRNAs in saliva differ across groups categorised by their hormone profiles at the point of concussion injury, 3) assess whether self-reported symptoms differ among groups categorised by their hormone profiles at the point of concussion injury, and 4) investigate whether low energy availability changes across the recovery period, and if this is associated with symptoms experienced.

### Study design

The prospective cohort study design involves recruitment of participants from Emergency Departments (ED) in Wellington, New Zealand.

### Patient and public involvement

Patients and members of the public were involved in the choice of outcome measures and the recruitment strategy in development of this research protocol. During the study design women with and without experience of a brain injury provided input on the feasibility and participant burden associated with each outcome measure through early experimental work and through informal discussion. The recruitment strategy and dissemination plan were refined based on discussions with patients presenting to ED with suspected concussion.

#### Participants and recruitment

**Fig. 1:**
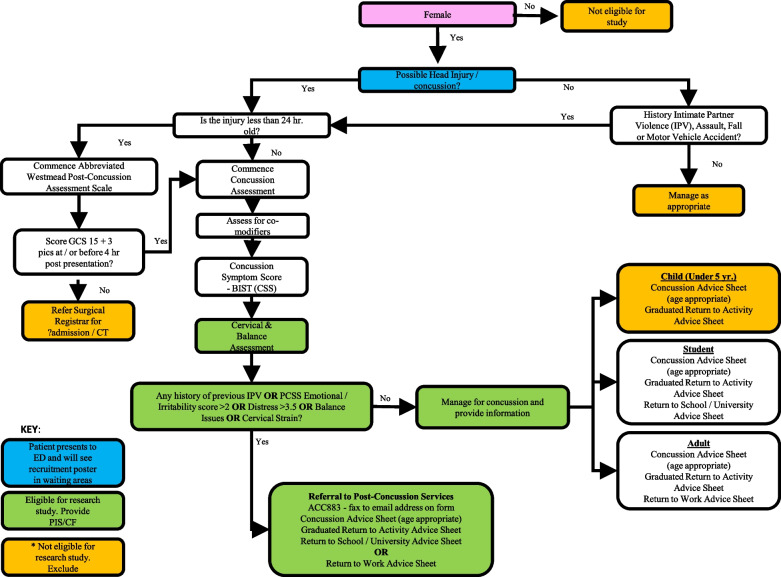
Routine clinical assessment and study flow

Females presenting to ED within three days of injury with suspected concussion are recruited by ED healthcare professionals. Participants are invited to participate if they are biologically female (self-report), of reproductive age (16–50 years) and have a confirmed diagnosis of concussion from the consulting healthcare professional. The diagnostic criteria for concussion are those outlined in the most recent international consensus statement from the concussion in sport group. [51] Participants will be excluded if they have Polycystic Ovarian Syndrome (PCOS), a pre-diagnosed psychiatric disorder or neurological condition (e.g. epilepsy, cerebral palsy) or if they have had more than three previously diagnosed concussions; these are known modifiers for prolonged recovery. [52, 53] These specific exclusion criteria will be ascertained by the consulting ED healthcare professional as part of the initial routine assessment for concussion. Full inclusion/exclusion criteria are shown in Table 1. The standard concussion assessment is detailed in Fig. 1. Study recruitment commenced February 2022, however there was interruption due to COVID related sickness and changes in the ED through November 2022. The study recommenced in January 2023 and is in progress.

**Table 1:** Inclusion and exclusion criteria

Inclusion criteria	Exclusion criteria
Females of reproductive age: 16–50 years (with a menstrual cycle for a minimum of 2 years if naturally cycling)	Had three or more previously diagnosed concussions
Natural regular menstrual cycle 28–35 days long, or Currently taking Oral Contraceptive Pill (OCP), or Currently have Intra Uterine Device (IUD), or	Current concussion was more than 3 days ago
	Post-menopause
	Started taking medication that would alter reproductive hormone concentrations (Corticosteroids, e.g., Prednisone. Antidepressant or Antipsychotic medication) within the last 3 months
	Current clinical diagnosis of an eating disorder
	Pre-diagnosed psychiatric disorder
	Pre-diagnosed neurological condition
	Polycystic Ovarian Syndrome (PCOS)
	Oligomenorrheic (irregular periods), or
	Amenorrheic (loss of periods for 3 months or longer)
Confirmed diagnosis of concussion by a medical doctor (within 3 days of injury)	GCS^a^ score less than 15 at 4 h after initial assessment. These patients may be referred for a CT scan and will not be eligible for the study

In the context of acute brain injury scores of < 8 = Severe, 9–12 = moderate, 13–15 = mild

^a^*GCS* Glasgow coma scale is used to objectively describe the extent of impaired consciousness in all types of acute medical and trauma patients

All participants will be over the age of 16 years and can therefore provide their own consent after reading a participant information sheet about the project. Participants will have the opportunity to ask questions of the research team prior to providing consent. Upon providing written consent, participants will be assigned a unique identifier number (e.g., CH001). If a healthcare professional advises that a patient may have temporary incapacity to provide consent due to the brain injury being treated at the ED, consent will be re-confirmed via follow up with the participant within three days using the contact information already provided on the consent form. Interpersonal violence (IPV) disproportionately affects females, to ensure safeguarding of any participants in this study that may be vulnerable the Te Whatu Ora (Health NZ) Family Violence Guidelines are implemented. [54] It is important that patients presenting to ED with injuries from IPV, assault or motor vehicle accidents are included as potential participants; concussion may often go unidentified due to polytrauma or an unrecognised mechanism of concussive injury. A specific concussion biomarker may be of most value in this cohort.

#### Sample size calculation

Using PASS15 software, and a Cox Proportional-Hazards regression model it was estimated that the number of participants needed to examine the primary aim (recovery time in days) is 30. This will allow the detection a hazard ratio of 1.5 (log hazard ratio coefficient = 0.4055), with 80% power, and a type I error rate of 0.05. This was calculated assuming a covariate SD of 1.5, a multiple regression R^2^ (variable of interest regressed on covariates) of 0.2, and an expected event (clinical recovery) rate of 0.9 within the follow-up period. [16, 22] Allowing for 20% drop out during follow-up, 38 participants will be recruited. As of 18 August 2023, 14 eligible women have started the study.

### Procedures and measures

Data collected from participants includes two saliva samples and two different surveys administered online weekly until the participant reports a full return to work/study.

#### Saliva samples

Participants will be asked to provide a 2 ml saliva sample. The sample, will be used to measure sncRNAs, and will be collected in SpeciMax stabilised saliva collection kits (Thermo Scientific SpeciMAX stabilised Saliva Collection Kit A50697, Life technologies Corp, Austin, Texas USA) which are pre-filled with 1 ml of virus inactivating and nucleic stabilization solution. Tubes are pre-labelled with a unique barcode linked with each participant’s unique identifier number. All samples are collected using a passive drool method. [55] Before providing each sample, participants take a small sip of water, swish the water around in their mouth and then swallow the water. This stimulates saliva release and clears any food debris that may be in the mouth. The participant’s approximate last mealtime is noted as this may influence saliva sample quality. The saliva sample is immediately stored in the freezer at − 80 °C until all samples are collected. Samples are then couriered on dry ice to laboratories in Auckland for processing and analyses.

#### Saliva sample analyses

On the day of analysis all samples will be brought to room temperature. Processing of saliva samples will involve 500 μL of each sample being aliquoted into an Eppendorf tube and centrifuged at 2,500 rpm for 5 min until all the proteins pelletise and the supernatant is clear.

#### sncRNA (miRNA) expression analysis

The sncRNA targets from saliva will be extracted using the MagMAX mirVana total RNA isolation kit (A27828, Thermo Fisher Scientific, Waltham, Massachusetts, US) using manufacturer’s instruction with modification for saliva extraction. After isolation of total RNA (including snc/miRNA), these will undergo polyadenylation, adapter ligation, reverse-transcription and miR-Amp reactions using the TaqMan™ Advanced miRNA cDNA Synthesis Kit (A28007, Thermo Fisher Scientific, Waltham, Massachusetts, US) following manufacturer’s instructions. Reactions will be performed on the standard thermocycler BioRad. The cDNA will be diluted, and real-time PCR runs for miRNA analysis will be performed using the Taqman Fast Advanced Mastermix for qPCR (4,444,964, Thermo Fisher Scientific, Waltham, Massachusetts, US) on QuantStudio™ 7 Flex Real-Time PCR System (Thermo Fisher Scientific, Waltham, Massachusetts, US).

Using TaqMan® Assays for miR-27a-5p and miR-30a-3p (assays 002445 and 000416 respectively, Thermo Fisher Scientific, Waltham, Massachusetts, US) their respective expressions will be calculated by the 2^−ΔΔCt^ method by using endogenous miRNA, one of RNU48, RNU44, U47, or RNU6B (Thermo Fisher Scientific, Waltham, Massachusetts, US) will be used as the reference genes. The ratio of miR-27a-5p/miR-30a-3p expression will be calculated. The ratio of these two miRNAs has previously been reported to differentiate concussed and non-concussed individuals; concentrations of miR-27a-5p and miR-30a-3p differentiated 75 concussed individuals from 97 non-concussed individuals with 82.4% sensitivity and 73.3% specificity. The levels of miR-27a-5p/miR-30a-3p were lower among concussed individuals and importantly showed no effect of acute or chronic exercise. These miRNAs showed similar accuracy for both sport and non-sport related concussion.

#### Surveys and questions

Survey and question data are collected and managed using REDCap (Research Electronic Data Capture) which is a secure, web-based software platform designed to support data capture for research studies. [56, 57] Participants are provided with an online link to REDCap via email. A new link is sent every seven days after initial appointment until a full return to work/study is reported. It takes approximately 10 min to complete all questions each week.

Two concussion specific questions are asked only once, seven days after the initial appointment. The first question relates to concussion history and is utilised to capture the number of previous concussions the participant has had. This information is important as prior concussion can impact recovery from subsequent concussions. [52] The second question is used to capture whether the participant’s concussion is sport related or not.

Participants are also asked to complete the Low Energy Availability in Females Questionnaire (LEAF–Q) online seven days after initial assessment and then at seven-day time intervals throughout recovery. The LEAF-Q [58] is designed and validated to evaluate physiological symptoms of insufficient energy intake or low energy availability (LEA). The LEAF-Q contains questions regarding injuries, gastrointestinal and reproductive function. In Sect. 3.2 three questions (19 items) relate to menstrual function including recent menstrual history. This questionnaire section is used to determine hormonal profile of participants and has been modified as outlined below.

Part C of Sect. 3.2 includes the following question:

- C: Do you have normal menstruation? Yes ☐ No (go to question C6) I don’t know (go to question C6)
- C1: If yes, when was your last period? 0–4 weeks ago ☐ 1–2 months ago ☐ 3–4 months ago ☐ 5 months ago or more ☐

This question has been modified to capture more accurate detail about the last menstrual cycle as follows:

- C: Do you have normal menstruation? Yes ☐ No (go to question C6) I don’t know (go to question C6)
- C1: If yes, when was your last period? 1 week ago ☐ 2 weeks ago ☐ 3 weeks ago ☐ 4 weeks ago ☐ 1–2 months ago ☐ 3–4 months ago ☐ 5 months ago or more

Participants are categorised into one of five hormone profiles as follows; 1) Follicular phase if question C1 is answered as “1 week ago”, 2) Mid-cycle if question C1 is answered “2 weeks ago”, 3) Luteal phase if question C1 is answered “3 weeks ago” or “4 weeks ago”, 4) Oligomenorrheic if C1 was answered “1–2 months ago”, “3–4 months ago” or “5 months ago or more”, 5) Using contraceptive if question 3.1a is answered “yes”.

Scoring of question 3.2 remains the same, i.e., if a participant ticks any of the boxes within the 0–4 weeks’ timeframe the same score is applied as the original question. In eumenorrheic participants this will help estimate menstrual cycle phase at point of injury. This information is used to understand any association between hormonal profile and or LEAF_Q score with symptom severity and duration of concussion.

The Brain Injury Screening Tool (BIST) is a brief screening tool for use on initial presentation after injury to guide health care pathway decision making and to monitor symptoms and recovery over time. [59] The BIST was developed in the NZ context to fit the clinical environment and to provide consistency across multiple environments and referral pathways (e.g. GP clinic, ED, Urgent Care Centres), the tool has also been psychometrically tested. There are two sections in the BIST; the first section is used to determine if there are any clinical ‘red flags’ which require referral to hospital, therefore this component is not included in this study. The second section of the BIST includes a 16-item symptom report scale for patients to rate how much they now experience the symptoms listed on a scale of 0 (not at all) to 10 (severe). Higher symptom scores are indicative of higher symptom burden. Participants are asked to complete this part of the BIST at point of presentation with injury and at seven-day time intervals throughout the study to monitor symptoms and recovery over time.

The final question asks if the participant has made a full return to work/study and how many follow-up appointments they had. The number of days between injury and full return to work/study will be used as a proxy for recovery. This question is included in the weblink sent at all timepoints (i.e., 7, 14, 21, 28 days etc.) until the participant reports they have achieved a full return to work/study, at which point participation in the study will be complete. Detail of information collected each week is included in Table 2.

**Table 2:** Research measures and collection points

Measure	How	Who	When		
			Initial assessment	During recovery (7-day time intervals)	Full return to work/study
Saliva Samples	Passive drool into cryovial	Consulting clinician, research assistant or Lead Researcher	X		
Number of prior concussions^b^	Online^a^	Participant to complete		X	
Sport or non-sport related concussion^b^	Online^a^	Participant to complete		X	
LEAF-Q	Online^a^	Participant to complete		X	
BIST	Online^a^	Participant to complete	X	X	
Number of days from injury to recovery (full return to work/study)	Online^a^	Participant to complete			X

^a^Weblink emailed to participant

^b^Question only asked once, at first (7-day) timepoint

## Data analysis

In this study a range of statistical tests will be used to analyse the data corresponding to the four aims. Aim 1 will use Cox proportional hazards regression to determine if the time to recovery from concussion (measured in days), differs among groups categorised by hormone profile at the point of injury (five profiles: Follicular phase, Midcycle, Luteal phase, oral contraception, IUD). Aim 2 will apply an ANOVA to compare the ratios of miR-27a-5p/miR-30a-3p expression, across these five groups. Model-estimated means and pairwise contrasts between groups will also be computed. For Aim 3, the focus is on whether self-reported symptoms, quantified both as the number of symptoms present (range 0–16) and as a total symptom burden (sum of severity scores; range 0–160), differ among hormone profile categories. These dependent variables will be analysed using ANOVA, with the five hormone profiles specified as the independent variable. Aim 4 will employ a linear mixed model to investigate changes in low energy availability across the recovery period. This model will incorporate the day of recovery and total symptom burden as fixed effects and will account for repeated measures over time by specifying participant as a random effect. The interaction between symptom burden and recovery time will also be evaluated to understand if these variables are collectively associated with low energy availability.

### Dissemination plan

A study fact sheet will be provided on our website https://sprinz.aut.ac.nz/areas-of-expertise/sports-kinesiology-injury-prevention-and-performance/female-athlete-performance-and-health-research-programme. Data will be available upon request from the primary author. Study findings will be published in peer-reviewed scientific journals and presented at relevant conferences. Participants will receive a summary of their individual results, written for a non-clinical audience. Participants will be informed of the full study results through the university website (https://sprinz.aut.ac.nz/areas-of-expertise/sports-kinesiology-injury-prevention-and-performance/female-athlete-performance-and-health-research-programme).

## Discussion

There is a lack of female specific sports science and biomedical research. [20, 60–65] Previous studies investigating candidate biomarkers for concussion have either been conducted in exclusively male cohorts or have not considered the influence that fluctuations in circulating sex hormones may have on the expression of salivary biomarkers. This protocol is important as it will help to determine whether females with a suspected concussion have similar salivary sncRNA expression to those reported in the literature for male athletes [35] and highlight any potential for false negative or positive results when considering salivary biomarkers as a diagnostic tool for females with suspected concussion. If this study confirms presence of sncRNAs in females with concussion, it will provide evidence of the potential of saliva sampling as an objective tool to aid in diagnosis of and confirmation of recovery from concussion. Findings will highlight whether expression and ratio of two sncRNAs may be associated with sex hormones in females and therefore whether this would be a feasible and reliable tool for routine clinical use in this cohort. Findings will also highlight how different hormone profiles may be associated with concussion symptom severity and duration in females.

### Strengths and limitations of the study

This is the first study to consider the impact of fluctuating female sex hormones on salivary sncRNA expression following concussion. Limitations of this study are that it relies on self-report hormone profiles and does not include verification using the ‘gold standard’ approach [66]. Menstrual cycle information is based on recall which can be subject to bias and inaccuracy. Return to work is being used as a proxy for recovery from concussion which does not necessarily reflect full physiological recovery. Information regarding treatment interventions and follow up appointments for each participant will not be collected, it will therefore not be known how the ongoing management may influence the recovery time. One further limitation could the relatively small sample size compared the number of variables under investigation. The power calculation is based on the primary aim of the study.

## Data Availability

No datasets were generated or analysed during the current study.

## Abbreviations

sncRNAs: Short non-coding Ribonucleic Acids
miRNAs: Micro Ribonucleic Acids
ED: Emergency department
